# Bioactive Compounds and Cardiac Fibrosis: Current Insight and Future Prospect

**DOI:** 10.3390/jcdd10070313

**Published:** 2023-07-21

**Authors:** Abdul Majid, Fasilat Oluwakemi Hassan, Md Monirul Hoque, Joy Olaoluwa Gbadegoye, Djamel Lebeche

**Affiliations:** 1 Department of Physiology, College of Medicine, The University of Tennessee Health Science Center, Translational Research Building, Room 318H, 71 S. Manassas, Memphis, TN 38163, USA; amajid1@uthsc.edu (A.M.); dhassanf@uthsc.edu (F.O.H.); mhoque5@uthsc.edu (M.M.H.); jgbadeg1@uthsc.edu (J.O.G.); 2 College of Graduate Health Sciences, The University of Tennessee Health Science Center, Memphis, TN 38163, USA

**Keywords:** cardiac fibrosis, cellular mechanisms, bioactive compounds, treatments

## Abstract

Cardiac fibrosis is a pathological condition characterized by excessive deposition of collagen and other extracellular matrix components in the heart. It is recognized as a major contributor to the development and progression of heart failure. Despite significant research efforts in characterizing and identifying key molecular mechanisms associated with myocardial fibrosis, effective treatment for this condition is still out of sight. In this regard, bioactive compounds have emerged as potential therapeutic antifibrotic agents due to their anti-inflammatory and antioxidant properties. These compounds exhibit the ability to modulate fibrogenic processes by inhibiting the production of extracellular matrix proteins involved in fibroblast to myofibroblast differentiation, or by promoting their breakdown. Extensive investigation of these bioactive compounds offers new possibilities for preventing or reducing cardiac fibrosis and its detrimental consequences. This comprehensive review aims to provide a thorough overview of the mechanisms underlying cardiac fibrosis, address the limitations of current treatment strategies, and specifically explore the potential of bioactive compounds as therapeutic interventions for the treatment and/or prevention of cardiac fibrosis.

## 1. Introduction

**Figure 1:**
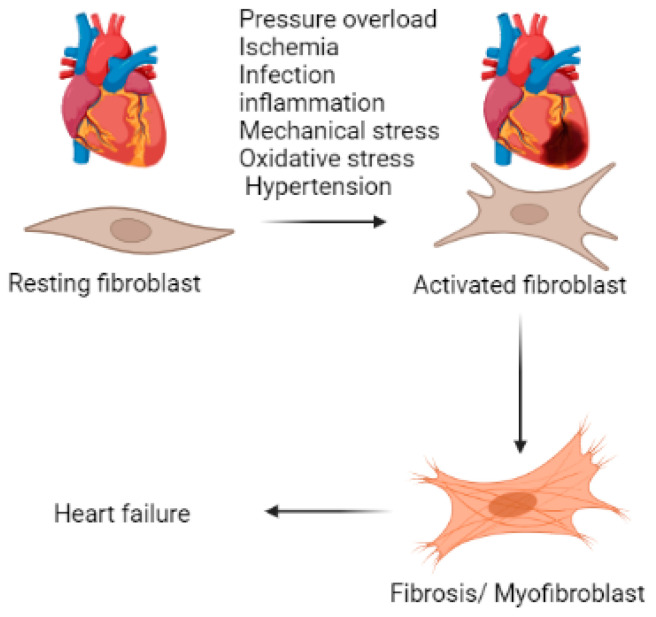
Factors involved in the development of fibrosis. Shown are the various stimuli that play a role in the activation of resting fibroblast, and fibroblast-to-myofibroblast differentiation, which results in cardiac fibrosis leading to chronic heart failure.

Cardiac fibrosis is a major contributor to cardiovascular diseases which claim nearly 18 million deaths annually [1]. Prolonged deposition of extracellular matrix (ECM) by activated cardiac fibroblasts, and the ensuing phenotypic conversion of fibroblasts to myofibroblasts, results in the loss of heart plasticity and reduction in contractility, often causing irreversible damage to the affected heart tissue [2,3]. Cardiac fibrosis could occur in response to a variety of stimuli such as inflammation, oxidative stress, ischemia, pressure overload, neurohumoral activation, cytokines and mechanical stress [4] (Figure 1). Traditionally, cardiac fibrosis has been described to have two main forms: reparative/replacement fibrosis, and reactive fibrosis. Replacement fibrosis involves the replacement of necrotic, severely inflamed, or damaged cardiac tissue following acute cardiac injury; this is seen, for instance, following myocardial infarction [5,6]. Reactive fibrosis usually occurs as a reaction to changes in myocardial load, and this form of fibrosis typically occurs in the absence of cell loss in the myocardium [7,8,9]. Reduced cardiac output, risk of arrhythmia, myocardial stiffness, diastolic dysfunction, and the development of heart failure are major abnormalities associated with pathological fibrotic remodeling and could ultimately result in sudden death of the affected patients [8].

A viable and efficient treatment for cardiac fibrosis remains elusive. While available drugs can alleviate the symptoms of cardiac fibrosis, as will be discussed below, there is a need for new agents that can specifically target the intracellular mechanisms underlying fibrosis. To address this urgent challenge, researchers are exploring various approaches to identify and develop new drugs for treating fibrosis. For example, some investigators focus on identifying novel molecular targets involved in fibrosis development and then designing drugs that can modulate different targets [9,10,11,12]. Others are exploring bioactive compounds and plant extracts that may have antifibrotic properties [13,14,15], aiming to isolate and characterize specific molecules that could be used as the basis for the development of new drugs that overcome the side effects caused by the currently available medications. 

In this review, we will look at the significance of related pathways during cardiac fibrosis, how they are involved in the existing and upcoming bioactive intervention, and which bioactive targets are promising for basic and applied cardiac research. To the best of our knowledge, there is a dearth of recent literature reviews that have comprehensively explored the role of bioactive compounds in cardiac fibrosis, with the exception of the limited review by Zhang et al. [13]. The scarcity of publications addressing this specific area highlights the need for further investigation and for a comprehensive updated review to serve as a guide for researchers in the field. Overall, while there is still much work to be done, there is reason to be hopeful that new and more effective treatments for cardiac fibrosis will be developed in the coming years.

## 2. Cellular and Molecular Mechanisms of Myocardial Fibrosis

**Figure 2:**
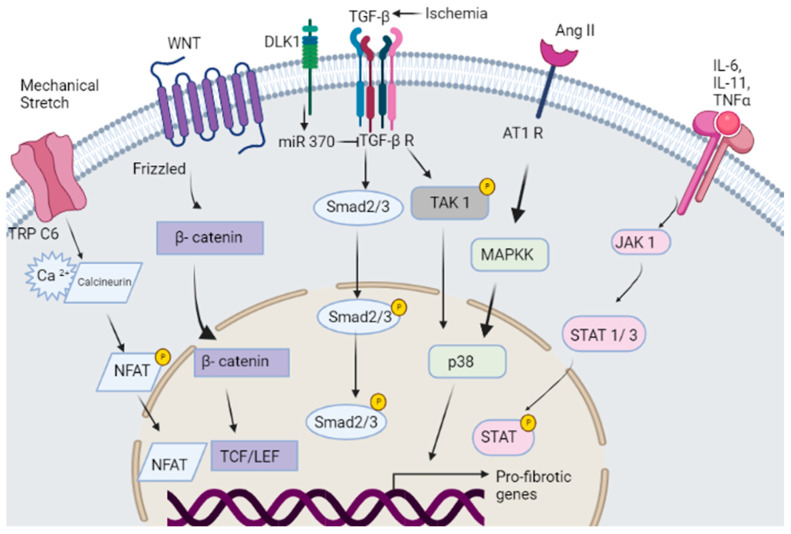
Signaling axes in cardiac fibrosis. Multiple stimuli have been demonstrated to orchestrate the activation of cardiac fibroblasts triggering the transcription of pro-fibrotic genes and fibrotic response. (1) Cytokines as IL1, IL2, TNFα though the activation of JAK1/STAT pathway; (2) Neurohumoral signaling via AngII binding to its membrane AT1R initiating pro-fibrotic gene transcription through MAPKK/p38 signaling; Ischemia-induced canonical and noncanonical TGFβ/TGFβR signaling via Smad2/3 or TAK1/p38; injury-induced Dlk1 attenuation of TGFβ/TGFβR2 signaling via activation of miR-370. Canonical WNT/β-catenic initiating gene transcription through activation of TCF/LEF signaling. Mechanical stretch, through stretch-sensitive ion channels and TRPC6 activate Ca^2+^-dependent calcineurin–NFAT signaling pathway to induce myofibroblast gene transcription.

Cardiac fibrosis involves complex cellular and molecular mechanisms that result in the deposition of ECM proteins, especially collagen, in the cardiac tissue [4]. The ECM, fibroblasts, myofibroblasts, immune cells and endothelial cells individually and collectively regulate the microenvironment of the fibrotic heart [16]. Dysregulation of these molecular interactions triggers the proliferation and activation of myofibroblasts leading to fibrotic responses which ultimately result in cardiac tissue maladaptive remodeling and dysfunction [17] (Figure 2).

Recent systems genetics studies have reported that there are approximately 275 genes related to cardiac fibrosis. Some of these genes have established roles in fibrosis, while some need more exploration to reveal their roles [18]. Identifying and understanding their roles and mechanisms will help devise clinical interventions for cardiac fibrosis.

Fibroblast activation involves the stimulation of the transforming growth factor-beta (TGF-β)/SMAD3 signaling pathway, the angiotensin II (Ang II) signaling pathway, the connective tissue growth factor (CTGF) signaling pathway, TAK/p38 MAPK (mitogen-activated protein kinase), Wnt/β-Catenin, G-protein-coupled receptor kinase (GRK), and Hippo signaling, among others [19] (Figure 2). Novel genes and nontraditional pathways are being reported along with ongoing studies on the well-known fibrotic genes in cardiac fibrosis, which contribute to myocardial fibrosis. For instance, Delta-like 1 homologue (Dlk1)*,* a paternally imprinted gene that encodes a transmembrane glycoprotein belonging to the Epidermal Growth Factor (EGF)-like family, has recently been documented to inhibit fibroblast-to-myofibroblast transdifferentiation and attenuates fibrosis [20]. Cytoskeleton-associated protein 4 (CKAP4), a stable and multifunctional transmembrane protein predominantly localized to the rough endoplasmic reticulum (RER), has been reported to play a role in the regulation of cardiac fibrotic remodeling. It is positively correlated to fibroblast markers in the human ischemic heart and identified as a novel marker for activated fibroblasts [21]. CKAP4 expression was upregulated specifically in activated ventricular fibroblasts, and its level increased in ventricular tissue following ischemic injury [21]. Other studies have also demonstrated that overexpression of CKAP4 significantly elevated the cell proliferation and migration of atrial fibroblasts, and it enhanced the levels of collagen I, fibronectin, MMP-1, and TIMP-1, while CKAP4 siRNA reduced them significantly [22]. Moreover, CKAP4 overexpression increased TGF-β1, c-jun, and c-fos expression significantly, and activated the JNK/p38 pathway [22]. 

Recently, the involvement of noncoding RNAs (ncRNAs) in the regulation of cardiac fibrosis has emerged. Ongoing studies have extrapolated the great potential of these RNAs in the diagnosis, prevention, intervention, and treatment of cardiac fibrosis. Several fibroblast-enriched and nonfibroblast microRNAs (miRNAs) that either inhibit or induce fibrosis directly or indirectly have been identified and validated [23,24,25,26,27]. We have, for instance, demonstrated that miR-1 overexpression in the heart of a rat model of pressure overload led to marked regression of left ventricular hypertrophy and myocardial fibrosis via targeting fibullin-2, a secreted protein implicated in ECM remodeling [28]. Similarly, we have shown that miR-370 is downregulated in ischemic human and porcine hearts and mediated Dlk1 attenuation of myocardial fibrosis [20]. miR-370 overexpression directly targets TGFβ-receptor II and attenuates TGFβ-1/Smad-3 profibrotic signaling [20]. miR-21 has been increasingly shown to regulate cardiac fibrosis: it stimulates fibroblast survival and growth factor secretion and induces cardiac fibrosis through activation of the ERK–MAPK activity and inhibition of Sprouty homologue 1 (Spry1) [29]; it activates the TGF-β/Smad pathway via suppression of TGF-β receptor III and facilitation of fibroblast–myofibroblasts differentiation following myocardial infarction (MI) injury [30], or regulates MMP-2 expression in fibroblasts and promotes the progression of fibrosis via inhibition of phosphatase and tensin homologue (PTEN) upon ischemia–reperfusion [31]. Inhibition of miR-21 under profibrotic conditions with AngII prevented fibrosis by increasing PTEN and SMAD7 [32]. Another miRNA, miR-125b, was found to be upregulated in both fibrotic human heart tissue and mouse models of cardiac fibrosis and was demonstrated to induce fibroblast-to-myofibroblast transition by functionally targeting apelin, a critical repressor of fibrogenesis [33]. Other key miRNAs involved in the cardiac fibrotic response are addressed in more detail elsewhere in other reviews [27].

Additionally, other types of ncRNA molecules, such as long noncoding RNAs (lncRNAs), circular RNAs, and small nucleolar RNAs have recently been described in the heart. Genome-wide profiling of cardiac tissue following MI has led to the identification of diverse lncRNAs differentially expressed in infarcted regions of the heart and remote myocardial tissue [34,35], although only a few have been experimentally validated. For example, the lncRNA Meg3 has been shown to be highly enriched in cardiac fibroblasts and promotes myocardial fibrosis and diastolic dysfunction in pressure overload mouse model by targeting MMP2 [36]. Silencing of Wisper (Wisp2 super-enhancer-associated RNA), a cardiac fibroblast-enriched lncRNA, attenuated MI-induced fibrosis and cardiac dysfunction [37]. Knockdown of MI-triggered upregulation of endogenous MIAT (myocardial infarction-associated transcript) reduced cardiac fibrosis and improved cardiac function via the regulation of the miR-24/furin-TGFβ-1 pathway [38]. Safe is a nucleus-enriched lncRNA that plays a role in cardiac fibrosis. Safe-Sfrp2-HuR complex-mediated stability of Sfrp2 mRNA was identified as an underlying mechanism of Safe-regulated myocardial fibrosis [34]. MALTA1 (metastasis-associated lung adenocarcinoma transcript 1) expression is increased in post-MI ischemia-reperfusion injury [39,40]. Its overexpression regulates TGF-β1 activity by manipulating the miR-145/Bnip3 pathway and promotes cardiac fibrosis [39,40]. Also, the direct interaction of a profibrotic lncRNA PCFL (pro-cardiac fibrotic lncRNA) with miR-378 alleviates the inhibitory effect of miR-378 on GRB2 (growth factor receptor-bound protein 2) and promotes post-MI fibrosis in the heart [41]. 

Sequencing of circRNA found a total of 283 circRNAs being expressed in the fibroblasts of fibrotic hearts, with 79 upregulated and 204 downregulated [42]. They were related to different cardiac fibrogenic signaling pathways; for example, TGF-β, AMPK, MAPK, and PI3K-Akt signaling pathways. circNFIB upregulation reduces cardiac fibrosis by sponging miR-433 in post-MI mouse model [43]. Upregulation of circHelz promoted cardiac fibrosis by binding to and facilitating the YAP1 (Yes-associated protein 1) localization in the nucleus [44]. On the other hand, antifibrotic circYap was shown to attenuate cardiac fibrosis by binding the tropomyosin-4 and gamma-actin complex [45].

## 3. Epigenetic Regulation of Cardiac Fibrosis

Recently, epigenetic regulation has emerged as a critical mechanism involved in the pathogenesis of cardiac fibrosis [46,47,48,49]. Epigenetic modifications, including DNA methylation and various histone modifications, can modulate gene expression without changing the underlying DNA sequence and thus play an essential role in regulating fibroblast activation and ECM deposition.

### 3.1. DNA Methylation 

DNA methylation changes were found to be associated with cardiac fibrosis in rats, mice, and human hearts [48,50,51]. Genes involved in ECM remodeling, such as collagen type I and III, were reduced following DNA methylation inhibition [52]. TGF-β1-induced demethylation of collagen type I alpha 1 (COL1A1) promoter in cardiac fibroblast led to its increased expression [53]. Mice deficient in DNA methyltransferase 1 (DNMT1), an enzyme that catalyzes DNA methylation, were found to have reduced cardiac fibrosis and improved cardiac function [54,55]. DNMT1-mediated hypermethylation of α-SMA promoter inactivates TGFβ-induced α-SMA expression, potentially reducing fibroblast differentiation and cardiac fibrosis [56]^.^ Hypermethylation of suppression of cytokine signaling 3 (SOCS3) promoter via DNMT1 downregulates SOCS3 expression levels in diabetic hearts and stimulates fibroblast activation and myocardial fibrosis [57]. DNMT3A-mediated silencing of Ras-association domain family 1 isoform A (RASSF1) exacerbated cardiac fibrosis in male rats [58]. Pharmacological DNMT inhibition with RG108 agent attenuated cardiac fibrosis associated with pressure-induced cardiac hypertrophy [59]. 

Global DNA hypermethylation and increased expression of DNMT1 and DNMT3B were reported to regulate hypoxia-induced cardiac fibrosis and increased expression of collagen 1 and α-SMA [60]. In contrast, Vujic et al. reported exacerbation of cardiac fibrosis in DNMT3B knockout mice [61]. Increased methylation of RASAL1 promoter diminished RASAL1 expression and ultimately led to cardiac fibrosis [62]. Cardiopulmonary exercise in heart failure patients was reported to reduce cardiac fibrosis via hypermethylation of acyl-CoA dehydrogenase very long-chain (ACADVL) gene [63]. Furthermore, studies have shown that the promoter region of the genes encoding matrix metalloproteinases (MMPs), which are enzymes involved in ECM degradation, are targeted by DNA methylation. For example, hypomethylation of MMP9, MMP2, and CTGF, genes implicated in ECM fibrosis, significantly enhances their gene expression in human failing cardiac tissues [64,65]. These studies, alongside other investigations, demonstrate that a wide range of MMPs are regulated in a temporal and spatial manner during post-injury cardiac remodeling [66,67], suggesting that manipulating the activities of MMPs requires approaches that prevent the potential adverse effects of MMPs on ECM/collagen turnover.

### 3.2. Histone Modification That Regulates Cardiac Fibrosis

Histone modifications play a significant role in the regulation of gene expression and can have a profound impact on the development of cardiac fibrosis [47,48]. Various modifications, such as acetylation or methylation, can modify the structure of histones and thereby affect the accessibility of DNA to the transcriptional machinery, ultimately influencing gene expression patterns [48,68,69,70]. Acetylation of histone H3 lysine 9 (H3K9ac) increased in high-glucose-stimulated cardiomyocytes, and mouse hearts contributed to cardiac fibrosis [71]. Elevated levels of Ac-H3K9 were seen in a hypertensive murine model, and inhibition of Ac-H3K9 reversed hypertension-induced myofibroblast differentiation, cardiac fibrosis, and diastolic dysfunction [72]. Histone modification also affects the activity of signaling pathways involved in cardiac fibrosis. For example, histone deacetylases (HDACs) inhibition has been reported to suppress the activity of MMP2 and MMP9 and reduce ventricular structural remodeling, leading to better cardiac function [73]. Mocetinostat, an HDAC inhibitor, reversed cardiac fibrosis and cardiac myofibroblast activation [74]. Myofibroblasts deficient in the histone LSD1 (lysine-specific demethylase-1), also called KDM1A, led to reduced cardiac fibrosis and deactivation of the TGF-β pathway and fibrotic gene expression following pressure overload stress [75]. Furthermore, studies have shown that histone lysine demethylases KDM3A and KDM3C bind the Timp1-promoter, a biomarker with profibrotic function [76,77], and stimulate its transcriptional activity, leading to activation of cardiac fibroblasts and induction of myocardial fibrosis [71,78], while KDM5B suppresses the expression of the activating transcription factor 3 (ATF3), an antifibrotic regulator of cardiac fibrosis, and stimulates the TGF-β signaling pathway and the subsequent increased expression of profibrotic genes [79].

Histone methyltransferases (HMTs), which are responsible for adding methyl groups to histones, have also been implicated in cardiac fibrosis. Inhibition of HMTs has shown the potential to reduce fibrosis by altering the expression of fibrosis-related genes [80]. Considering the role of histone modifications in cardiac fibrosis, modulating these modifications through pharmacological or genetic approaches holds promise as a potential therapeutic strategy. Targeting specific enzymes involved in histone acetylation or methylation, such as HDACs or HMTs, may help restore proper gene expression patterns and prevent or reduce cardiac fibrosis.

## 4. Currently Available Antifibrosis Treatments

At present, there are no FDA-approved drugs for the treatment of cardiac fibrosis. This has partly been due to the inability to adequately quantify fibrotic burden by noninvasive methods such as echocardiography [8]. Other imaging methods such as the Magnetic Resonance Imaging, and late Gadolinium enhancement cardiac magnetic resonance are not clinically feasible for use in early detection and quantification of fibrosis in all patients [6]. However, some medications approved for other disease conditions have been recognized to have limited effect in treating clinical complications of cardiac fibrosis (Table 1).

TGF-β1 inhibitors: Pirfenidone, an FDA-approved medication for the treatment of idiopathic pulmonary fibrosis, has been shown to limit fibrotic expansion post-infarction in the heart but has no effect on the diastolic function, which is a major cause of complications associated with cardiac fibrosis [81]. The effect seen with pirfenidone could be related to inhibition of TGF-β signaling, which is critical in the induction of fibrotic remodeling [8]. Coleman et al. have described peptide of the N terminus of a G protein-coupled receptor Kinase 5 (GRK5nt) as a potential candidate for the treatment of cardiac fibrosis. However, associated complications include heart failure. This is due to its ability to bind calmodulin and block the calcium–calmodulin complex from binding to G protein receptor kinase after pressure overload, reducing the severity of pathological GRK5 signaling associated with heart failure [82]. This peptide exerts its effect by affecting the β adrenergic receptor system, which is critical in maintaining blood pressure via the GPCR/c-AMP pathway and helps prevent adverse cardiac remodeling associated with fibrosis.

Guanylate Cyclase Stimulators: Vericiguat, used in the treatment of heart failure with reduced ejection fraction (HFrEF) [83], has been shown to reduce myocardial ischemic injury in experimental animals and reduce fibrosis biomarkers in cardiac fibrosis in human patients. This drug increases sensitivity to endogenous nitric oxide, causing vasodilation and increased tissue perfusion. However, clinical studies show that vericiguat does not significantly reverse the adverse effects of fibrosis and has less effect on cardiac function [84].

Mineralocorticoid receptor antagonists: Spironolactone, a diuretic that inhibits aldosterone signaling in the treatment of patients with chronic heart failure, has been demonstrated to have some antifibrotic activity by reducing ECM turnover rate [85]. Spironolactone is also approved by the FDA for the treatment of HFrEF and resistant hypertension. In addition, treatment with spironolactone does not significantly increase ejection fraction but reduces fibroblast to myofibroblast differentiation [86]. However, further studies are required to understand the potential mechanisms mediating spironolactone activity in cardiac fibrosis.

Renin–Angiotensin–Aldosterone Systems (RAAS) inhibitors, such as lisinopril, an angiotensin-converting-enzyme (ACE) inhibitor, and losartan, an angiotensin II receptor blocker, are FDA-approved medications for the treatment of hypertension but also have been shown to reduce the synthesis of collagen, which is associated with cardiac fibrosis [87]. Losartan use in patients with nonobstructive hypertrophic cardiomyopathy were shown to significantly reduce the progression of cardiac fibrosis [87]. RAAS inhibitors inhibit the TGF-β profibrotic pathway to prevent fibrosis by restoring a balance to the ACE/ACE2 ratio, which is critical in the prevention/treatment of fibrosis [88]. However, more studies are needed to elucidate specific mechanisms at different stages of cardiac fibrosis that RAAS inhibitors could be effective.

Inflammation inhibitors: The use of TNFα inhibitors with anti-inflammatory effects in the prevention of cardiac fibrosis has also been documented by the RENEWAL and ATTACH trials; however, these were abruptly ended due to increased mortality in patients with chronic heart failure [89]. Several other anti-inflammatory medications, such as rosuvastatin, have been investigated in two major large-scale clinical trials, with no reported major effect on cardiac fibrosis and heart failure [90,91]. Activation of peroxisome proliferator-activated receptor α (PPAR-α) genetically or by agonists such as fenofibrate, has been shown to prevent inflammation and fibrosis [92,93,94,95,96]; however, the cardiac safety profile of these PPAR-α agonists has not been clearly elucidated [97,98]. They may in fact produce the opposite effect by exacerbating cardiac chamber dilation and including fibrosis [99].

Endothelin receptors inhibitors: For instance, Bosentan, an FDA-approved medication for the treatment of pulmonary hypertension, was initially seen to improve systemic hemodynamics in patients with severe heart failure on ACE inhibitors, but it unfortunately has a neutral effect in the treatment of cardiac fibrosis [100].

SKI, a homolog of the avian Sloan–Kettering virus in humans, is a known negative regulator of TGFβ1/Smad signaling and fibrotic response via suppression of the R-Smad function. SKI was experimentally found to inhibit fibrosis in two different models of heart failure by deactivating cardiac fibroblasts [101]. However, a need for further study to elucidate the role of SKI in clinical cases is necessary.

Histone deacetylase inhibitors: Zinc-dependent FDA-approved histone deacetylase pan inhibitor, vorinostat has been documented to play an antifibrotic role by inhibiting the transcription of collagen and α-SMA, but its molecular mechanism in inhibiting cardiac fibrosis is still unknown [102].

CAR-T cells: Chimeric Antigen Receptor (CAR)-T cells are engineered receptors that redirect T lymphocytes to recognize and eliminate cells expressing a specific target antigen [103,104,105]. Aghajanian et al. described the effectiveness of CAR-T cell infusion in fibrosis by elimination of fibroblast-activating protein, a process that was shown to be safe and less cardiotoxic [9,104]. The major challenges with this strategy are the long-term treatment required and the high cost associated with the treatment procedure; other reported adverse effects are the complications of cardiogenic shock and the associated multiorgan failure [105]. Further studies are needed to defer more effective and safer applications of CAR-T cells in the treatment of cardiac fibrosis [103].

Endogenous hormones such as relaxin have been shown to exert a strong vasodilation effect and inhibit cardiac fibrosis by inhibiting TGF-β [106] and Smad pathways; relaxin has been shown in various experimental models to exert an antifibrotic effect in the heart [107]. However, a clinical trial with relaxin alone has failed, in part due to its short half-life, hence the short duration of effect and its high cost implication [108]. Nevertheless, further studies on the use of relaxin with other classes of ACE inhibitors might be required to attempt to fully enhance the potential of relaxin in the treatment of cardiac fibrosis [109].

Other novel therapeutic approaches currently examined for cardiac fibrosis include the use of noncoding RNAs [9,26], microRNAs have been reported in several research studies to modulate cardiac fibrosis, miRNA binding to messenger RNA induces gene silencing, and genes involved in the progression or inhibition of cardiac fibrosis are often affected. However, the effect of miRNAs studied has been inconsistent, as downregulation of some miRNAs exaggerates collagen deposition, while silencing of some other miRNAs reduces collagen formation and cardiac fibrosis progression, as over 70 miRNAs have been associated with cardiac fibrosis pathophysiology. The initial clinical trial involving HF patients represents the first instance of examining an antisense drug. The drug, known as CDR132L (a specific antisense oligonucleotide, is a first-in-class miR-132 inhibitor), demonstrated safety and tolerability, as well as linear plasma pharmacokinetics without any indications of accumulation [110]. Furthermore, it indicates potential improvements in cardiac function. While the study’s limitation lies in the small number of patients, the drug’s promising efficacy strongly supports the need for further clinical investigations to confirm the beneficial pharmacodynamic effects of CDR132L in treatment [110].

**Table 1:** Therapies currently used in clinical practice.

Classes of Drugs	Effects	References
TGF beta inhibitors	Inhibit fibroblast to myofibroblast transformation. Approved for the treatment of idiopathic pulmonary fibrosis but do not improve diastolic function in cardiac fibrosis	[8,111]
Angiotensin-Converting Enzyme inhibitors (ACE inhibitors)	Collagen volume fraction reduction, side effect of hypotension in prolonged use.	[112]
Angiotensin Receptor Blockers (ARB)	Type I Collagen degradation, reduce infarct size	[9,112]
Mineralocorticoid Receptor Antagonist (MRA)	Decreases serum markers of cardiac fibrosis; prevents cardiac remodeling following MI. Side effects may include hypotension	[113]
Angiotensin Receptor neprilysin inhibitor	Treatment of HFrEF; prevents cardiac remodeling post-MI	[114]
Connective tissue Growth factor (CTGF) inhibitors	Attenuate development of fibrosis in idiopathic pulmonary fibrosis and currently in Phase 3 clinical trial	[115]
Hyperpolarization-activated cyclic nucleotide-gated (HCN) channel blockers	Reduction in serum aldosterone; reduce fibroblast activation	[116]
Matrix metalloproteinases inhibitors	Digest excessive ECM: reduce left ventricular remodeling and have an antibacterial effect; however, cause photosensitivity	[117,118]
Soluble guanylate cyclase (sGC) stimulators	Improve outcome in patients with HFrEF. Vericiguat is currently in a clinical trial (NCT05799638)	[119]
Beta 3-adrenergic receptor agonist	Improves ejection fraction of the left ventricle. However, causes increased blood pressure via the modulation of nitric oxide. Side effects include bronchospasm and depression	[120]
Hydroxymethylglutaryl coenzymeA Reductase Inhibitors	Reduction of atherosclerosis; anti-inflammatory effect; inhibit cardiac remodeling following MI. Side effect includes rhabdomyolysis	[121]

## 5. Challenges with the Development of Therapy for Cardiac Fibrosis

Much of the knowledge about cardiac fibrosis and its mechanisms emerged from experiments performed in cell-culture systems or global knockout mice. The lack of suitable in vivo markers of fibroblasts, their staging, and appropriate lineage mapping tools limit our ability to understand the regulatory process of cardiac fibroblast proliferation and differentiation into myofibroblasts following injury [111]. Along with these challenges, the fibrotic cascade involves multiple dynamic cross talks among pro-inflammatory and anti-inflammatory cell populations.

Another concern is that cardiac fibrosis works like a double-edged sword. Fibrosis plays an important protective role in preserving the structural integrity of the heart during the healing process and thus secures the organ from rupture after myocardial infarction. As a result, complete inhibition of fibrotic responses following myocardial infarction and other cardiac stresses might have harmful consequences [122]. However, the exaggerated fibrotic responses cause local or global deposition of excess collagen fibers in the myocardium, often resulting in pathological conditions [123]. Hence, one of the major challenges in treating cardiac fibrosis is to use the beneficial and cardioprotective role of fibrosing mechanisms while averting unwanted overreacting pathological remodeling. This requires adequate knowledge of what stage to target in therapy.

Although some novel treatments have shown promising results in in vivo and preclinical studies, we do not have approved therapies specifically targeting cardiac fibrosis. Some approaches have proved themselves to be potential candidates for antifibrosis therapy. Among them, genetic engineering of stem cells by silencing fibrosis-related genes with CRISPR technology [124]; targeting potential fibrotic genes and their expression with miRNA and epigenetic regulators, for example, HDACs and methyltransferases [49]; using antifibrotic medications, such as NLRP3 inflammasome inhibitors [125]; and delivering targeted therapy with CAR-T cells [126] are promising approaches. However, their application to address cardiac fibrosis is still limited.

One major loophole in the rationale for implementing such strategies is that they are often based on simplified concepts. Moreover, complex feedback regulatory loops make it challenging to draw inferences about homogeneous epigenetic modifications. However, cardiac fibrosis is not a singular disease but a cluster of pathologic abnormalities. The interventions in the animal models are commonly initiated at an earlier stage of a dynamic model of progressive fibrosis. However, patients with heart failure often present with a mature or advanced fibrotic lesion, which limits the translational value of this approach. On the other hand, specific pathways might need to be targeted chronically over a long period of time to halt or reverse the progression of myocardial fibrosis. Many of these pathways have integral roles in the protective and reparative functions in response to various injurious stimuli. Hence, negating the homeostatic roles of such pathways in an optimum way is challenging.

## 6. Bioactive Agents

### 6.1. Bioactive Compounds

**Figure 3:**
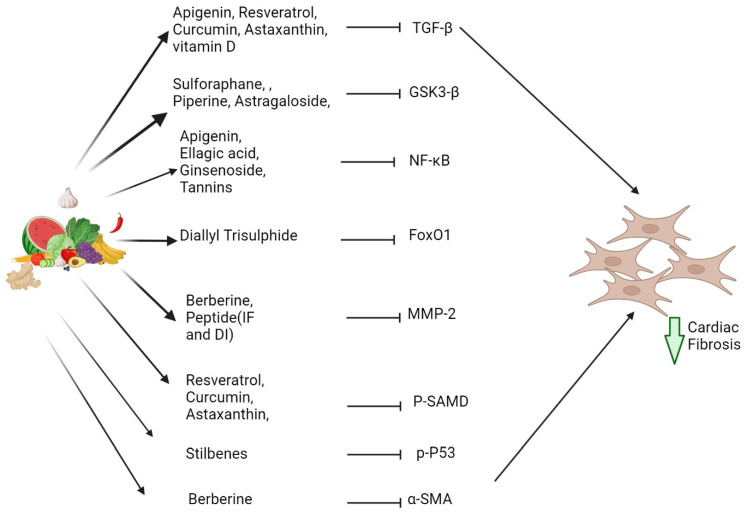
Attenuation of cardiac fibrosis by various bioactive compounds via distinct molecular targets.

Bioactive compounds are naturally occurring compounds that possess biological activity and have been investigated for their potential to prevent or reduce cardiac fibrosis. These compounds can be found in various plant-based foods and have been shown to possess anti-inflammatory and antioxidant properties [127]. Additionally, some bioactive compounds have been found to inhibit the production of ECM proteins and promote their breakdown, which can reduce the accumulation of collagen in the heart tissue by targeting various pro-inflammatory pathways. Polyphenols, including flavonoids, have been shown to reduce cardiac fibrosis by inhibiting the activation of fibroblasts, reducing collagen synthesis, and promoting collagen degradation. Resveratrol, a polyphenol found in grapes and red wine, has been shown to reduce cardiac fibrosis in animal models by inhibition of TGF-β [13] and reduce production of collagen in cardiac fibroblasts [128]. Similarly, genistein, a soy-derived isoflavone, has been found to inhibit the expression of TGF-β and reduce the production of collagen in cardiac fibroblasts [129]. In the following part of the review, we will explore examples of bioactive compounds (Table 2) for their potential to prevent or treat cardiac fibrosis and discuss the various molecular and cellular mechanisms regulated by these compounds (Figure 3).

#### 6.1.1. Flavonoids

Flavonoids are a group of bioactive compounds found in many plant-based foods, including fruits, vegetables, tea, and cocoa. They have been extensively studied for their health benefits, including their potential to prevent or treat various cardiovascular diseases, including cardiac fibrosis [130,131,132,133].

Quercetin: Quercetin is a flavonoid found in many fruits and vegetables, including onions, apples, and broccoli. Studies have shown that quercetin can inhibit cardiac fibroblasts producing collagen and other ECM proteins and reduce inflammatory actions, which may help prevent cardiac fibrosis [134,135]. One such study was carried out by Chang et al., in which they have proved that quercetin inhibits myocardial fibrosis by enhancing mitochondrial energy metabolism and managing the mitochondrial fusion/fission and mitochondrial biosynthesis while preventing the inflammatory response and oxidative stress injury [136].

- Epicatechin: Epicatechin is a flavonoid found in cocoa and tea. Studies have shown that epicatechin can improve cardiac function and reduce cardiac fibrosis in an aged, female rat model of pre-HFpEF [137].
- Kaempferol: Kaempferol, a flavonoid found in many fruits and vegetables, including grapes, broccoli, and kale, can inhibit the proliferation of cardiac fibroblasts and reduce collagen deposition in the heart, which may help prevent cardiac fibrosis [138].
- Apigenin: Apigenin, a flavonoid found in many fruits and vegetables, including parsley, celery, and chamomile tea, inhibits ECM proteins and collagen production in cardiac fibroblasts, which may help prevent cardiac fibrosis [139]. The study also showed that apigenin inhibits isoproterenol-induced myocardial fibrosis in mice via enhancing the antioxidant’s capacity to exert its antifibrotic effects, and it also decreases the NF-κB/TGF-β1 signaling pathway axis [140].

#### 6.1.2. Organosulfur Compounds

Organosulfur compounds are a group of bioactive compounds found in many foods, including garlic, onions, and cruciferous vegetables (such as broccoli, cauliflower, and kale). These compounds have been studied for their potential health benefits, including their potential to prevent or treat various cardiovascular diseases, including cardiac fibrosis [141]. The following are a few reported organosulfur compounds:

- Allicin: Allicin is an organosulfur compound found in garlic. Studies have shown that allicin can inhibit the proliferation of cardiac fibroblasts and reduce collagen deposition in the heart, thus preventing cardiac fibrosis [142,143].
- Sulforaphane: Sulforaphane is an organosulfur compound, which mainly exists in the form of a precursor called Glucorapahnin found in cruciferous vegetables. Sulforaphane can improve cardiac function and reduce fibrosis in animal models of heart failure [144,145]. Wang et al. showed that nuclear factor erythroid 2-related factor 2 (Nrf2) plays a pivotal role in protecting against Ang II-induced aortic fibrosis. Furthermore, sulforaphane inhibited Ang II-induced aortic damage by stimulating Nrf2 through the ERK/GSK-3β/Fyn pathway [146].
- Diallyl trisulfide: Diallyl trisulfide, an organosulfur compound found in garlic, has been shown to reduce the expression of genes involved in fibrosis and improve cardiac function in an isoproterenol-induced acute myocardial infarction rat model of heart failure. Diallyl trisulfide therapy demonstrated cardioprotective benefits via modulation of autophagy, PI3K/Akt signaling, eNOS, and FOXO-1 downregulation [147].
- S-allylcysteine: An organosulfur compound found in garlic, it can inhibit the proliferation of cardiac fibroblasts and reduce collagen deposition in the heart, which may help prevent cardiac fibrosis [148]. A study by Zainalabidin et al. also showed that S-allylcysteine reduces adverse cardiac remodeling after myocardial infarction in a rat model and also limits cardiac fibrosis in rats [149].

#### 6.1.3. Terpenoids

Terpenoids are a large and diverse group of naturally occurring compounds found in many plant-based foods, including fruits, vegetables, herbs, and spices. Terpenoids have been extensively studied for their potential health benefits to prevent or treat various cardiovascular diseases, including cardiac fibrosis. Several studies have investigated the effects of terpenoids on cardiac fibrosis [150].

- β-Caryophyllene: β-Caryophyllene is a terpenoid found in many herbs and spices, including black pepper, oregano, and cloves. Studies have shown that β-caryophyllene can shield the cardiac tissues against cardiotoxicity by mitigating inflammation, reducing collagen deposition, and improving cardiac function in rat models [151].
- Limonene: Limonene, found in many citrus fruits, including oranges, lemons, and limes, inhibits ECM proteins and the production of collagen in cardiac fibroblasts, which may help prevent cardiac fibrosis [152]. It can ameliorate cardiac injury induced by carbon tetrachloride intoxication through its antioxidant and anti-inflammatory potential [153].
- Ursolic acid: Ursolic acid, found in many fruits, vegetables, and herbs, including apples, cranberries, and rosemary, has shown that ursolic acid improves cardiac function in STZ-induced diabetic cardiomyopathy rats by attenuating inflammation and fibrosis [154,155].
- Rosmarinic acid: Rosmarinic acid is a terpenoid found in many herbs, including rosemary, sage, and thyme. Rosmarinic acid has been reported to reduce collagen deposition and improve cardiac function in animal models of heart failure. Supplementation of rosmarinic acid attenuated cardiac dysfunction, as well as inhibited cardiac fibrosis and prevented transdifferentiation of cardiac fibroblast [156].
- There are other terpenoids, such as Artemisinin, Betulin, Celastrol, Dioscin, Geniposide, Ginsenoside Rg3, Oridonin, Sweroside, Triptolide, and oleanolic acid, that are also involved in decreasing cardiac inflammation and myocardial fibrosis, improving left-ventricular function, and inhibiting NF-κB protein expression in rats with experimental diabetic cardiomyopathy [150,157]. This implies that these terpenoids have myocardial protection, which is related to their anti-inflammatory effects [150,157].

#### 6.1.4. Phenols

Polyphenols are a large and diverse group of naturally occurring compounds found in many plant-based foods, such as fruits, vegetables, whole grains, tea, and coffee. They are characterized by the presence of multiple phenolic groups, which are responsible for their antioxidant and anti-inflammatory properties. Polyphenols can scavenge free radicals and reduce oxidative stress. Oxidative stress causes inflammation and cardiac tissue damage. Furthermore, certain polyphenols have demonstrated the ability to decrease the expression of pro-inflammatory cytokines, which play a role in the development of cardiac fibrosis. Because of their anti-oxidant and anti-inflammatory actions, polyphenols, particularly those found in plant-based foods, may have protective effects against cardiac fibrosis [150].

- Resveratrol: Studies have demonstrated that resveratrol, a polyphenol found in grapes, red wine, and peanuts, can reduce fibrosis and inflammation in the heart [154]. Many studies found that resveratrol reduced cardiac fibrosis in rats with hypertension [158,159,160,161]. Resveratrol supplement reduced NLRP3 inflammasome activity, decreased TGF-β1 production, and downregulated the p-SMAD2/SMAD2 expression in a rat model of acute myocardial infarction, thus protecting the heart against cardiac fibrosis [162].
- Curcumin: A natural compound found in turmeric, a spice commonly used in Indian and Middle Eastern cuisine, has been shown to have anti-inflammatory, anti-apoptotic and antioxidant properties. There is evidence to suggest that it may have beneficial effects on cardiac fibrosis [163]. Several studies have investigated the effects of curcumin on cardiac fibrosis in animal models of cardiac disease. These studies have reported that curcumin can reduce the expression of profibrotic genes and proteins, as well as the deposition of collagen in the heart [164]. Curcumin has also been shown to decrease oxidative stress and inflammation, key factors in the development of cardiac fibrosis.
- Curcumin has been shown to protect the heart from cardiac fibrosis after myocardial infarction by inhibiting macrophage–fibroblast cross talk in the acute phase post-injury and retrained the activation of IL18-TGFβ1-p-SMAD2/3 signaling in the mice model [165]. Another study suggested that abemaciclib administration causes cardiac damage and increases cardiac fibrosis. However, co-administration of curcumin and abedmaciclib suppressed myocardial fibrosis associated with cardiac damage [166]. While these findings are promising, it should be noted that the majority of studies on the effects of curcumin on cardiac fibrosis have been undertaken in animal models, and further study is needed to determine whether curcumin has similar benefits in human subjects and what appropriate doses are needed in the treatment of cardiac fibrosis.
- Ellagic acid is a natural phenolic compound found in various fruits, nuts, and vegetables. It has been shown to have a range of potential health benefits, including anti-inflammatory, antioxidant, and anticancer effects. However, there is limited research on its effects on cardiac fibrosis. Ellagic acid has been reported to significantly reduce cardiac fibrosis in rats with myocardial infarction [167]. The effect of ellagic acid has been attributed to its antioxidant and anti-inflammatory properties, which can help to prevent the activation of fibroblasts and the production of ECM proteins.
- There are other polyphenols that are also involved in decreasing cardiac inflammation and myocardial fibrosis, improving left-ventricular function, and inhibiting NF-κB protein expression and NLRP3 inflammasomes in rat/mice models, including Ferulic acid, Gallic acid, Paeonol, Phloretin and Salidroside [150].

#### 6.1.5. Alkaloids

Alkaloids are a group of naturally occurring compounds found in many plant-based foods, including fruits, vegetables, herbs, and spices. Alkaloids have been extensively studied for their potential various cardiovascular health benefits. Several studies have investigated the effects of alkaloids on cardiac fibrosis. Here are some of the findings:

- Berberine: Berberine is an alkaloid found in many herbs, including goldenseal, barberry, and Oregon grape. Long-term berberine administration reduces cardiac fibrosis and dysfunction in diabetic rats by downregulating IGF-1R expression in cardiac fibroblasts and subsequently lowering MMP-2/MMP-9, α-SMA, and collagen type I expression [168].
- Vincamine: Vincamine is an alkaloid found in the leaves of the lesser periwinkle plant, which was found to reduce pathological cardiac remodeling by decreasing cardiac myocyte hypertrophy, fibroblast activation, and fibrotic gene expression. [169,170].
- Piperine: Piperine is an alkaloid mostly found in black pepper, has been demonstrated to offer various health benefits, including anti-inflammatory and antioxidant effects. There is some evidence to suggest that piperine may have potential in the treatment of cardiac fibrosis in animal models. One study found that piperine attenuates cardiac fibrosis via the activation of PPAR-γ and the resultant inhibition of AKT/GSK3β [171]. The mechanism by which piperine attenuates cardiac fibrosis is not fully understood, but it may be related to its anti-inflammatory and antioxidant properties.

Altogether, these studies suggest that alkaloids may have potential therapeutic benefits for preventing or treating cardiac fibrosis.

#### 6.1.6. Saponins

Saponins are a group of natural compounds found in a variety of plants, including soybeans, oats, and ginseng. Some studies suggest that saponins may have potential benefits for cardiac fibrosis [172].

- Ginsenoside: Ginsenoside is a triterpene saponin found in ginseng, which can ameliorate isoproterenol-induced myocardial fibrosis via regulation of the TGF-β1/Smad3 pathway [172]. Ginsenoside can ameliorate acute myocardial infarction and angiotensin Ⅱ-induced myocardial fibrosis. The mechanism is at least partially related to the regulation of the miR-489/myd88/NF-κB signaling pathway. Others have reported that treatment with ginsenosides reduced the deposition of collagen and improved heart function in mice [173].
- Astragaloside IV: Astragaloside IV is a significant active astragaloside component of Astragalus Propinquuos and is prominent in cardiovascular disease studies. Astragaloside IV may prevent myocardial infarction-induced fibrosis by inhibiting the endothelial-to-mesenchymal transition mediated by the AKT/GSK3-β/SNAIL signaling pathway [172]. While these studies suggest that saponins may have potential benefits for cardiac fibrosis, more research is needed to confirm these effects in humans.

#### 6.1.7. Coumarins

Coumarins are a group of natural compounds found in a variety of plants, including cinnamon, parsley, and chamomile. Some studies suggest that coumarins may have potential benefits for cardiac fibrosis, although more research is needed to confirm these effects [174,175]. The effects of scopoletin, a type of coumarin found in cinnamon, on cardiac fibrosis in rats has been reported to exhibit better therapeutic potential against isoproterenol-induced myocardial infarction in rats [176]. While these studies suggest that coumarins may have potential benefits for cardiac fibrosis in animal studies, more research is needed to confirm these effects in humans.

#### 6.1.8. Stilbenes

Stilbenes are a group of natural compounds found in a variety of plants, including grapes, peanuts, and berries. Treatment with pterostilbene has been shown to inhibit oxidative stress-mediated Pitx2c/miR-15b pathway and suppression of p-p53-dependent TGF-β1/Smads/CTGF activation, resulting in the alleviation of high-fructose-induced myocardial fibrosis [177]. While these studies suggest that stilbenes may have potential benefits for cardiac fibrosis, the side effects of stilbenes are also of great concern [150].

#### 6.1.9. Phenolic Acids

Phenolic acids are a group of natural compounds found in a variety of plants, including fruits, vegetables, and whole grains. Some studies suggest that phenolic acids may have potential benefits for cardiac fibrosis, although more research is needed to confirm these effects [150].

- Caffeic acid, a type of phenolic acid found in coffee and other foods, has an effect on cardiac fibrosis in rats. Treatment with caffeic acid reduced the deposition of collagen and improved heart function in rats [178,179].
- Chlorogenic acid: Chlorogenic acid is another type of phenolic acid found in coffee and other foods. Studies reported the antifibrotic function of chlorogenic acid in diabetic heart and established that chlorogenic acid exerted its antifibrotic effect through activation of the NO/cGMP/PKG pathway [180,181].
- Vanillic acid, which is a phenolic compound widely found in plants and fruits, has a protective role on right-ventricular function by inhibiting the Rho-associated protein kinase signaling pathway. It may also prevent cardiac fibrosis, promote cardiomyocyte enlargement, and prevent cardiomyocyte apoptosis in Rats with Monocrotaline-Induced Pulmonary Arterial Hypertension [182]. While these studies suggest that phenolic acids may have potential benefits for cardiac fibrosis, more research is needed to confirm these effects in humans.

#### 6.1.10. Anthocyanins and Tannins

Anthocyanins are a group of natural compounds found in a variety of colorful fruits and vegetables, such as blueberries, raspberries, and red cabbage. Some studies suggest that anthocyanins may have potential benefits for cardiac fibrosis. Anthocyanin was reported to have improved heart function in diabetic mice by reducing inflammation and fibrosis. Anthocyanin has anti-inflammatory and antifibrotic properties in high-glucose-induced cardiac fibroblasts via the interaction of miR-214-3p and IL-17 [183].

Tannins are a group of natural compounds found in a variety of plant-based foods and beverages, such as tea, coffee, and wine. The effects of Tannic acid, a type of tannin found in green tea and red wine, protected against myocardial fibrosis in isoproterenol-induced mice. This protection was achieved by suppressing the increases in NF-κB (P65), TLR4, p38 expression [184]. In addition, researchers found that treatment with grape seed tannins reduced the deposition of collagen and improved heart function in rats. While these studies suggest that tannins may have potential benefits for cardiac fibrosis, more research is needed to confirm these effects in humans.

#### 6.1.11. Carotenoids

Carotenoids are a group of naturally occurring pigments that are synthesized by plants and some microorganisms. They are known for their antioxidant properties and are commonly found in fruits and vegetables, particularly those that are yellow, orange, or red in color [185]. There is some evidence to suggest that carotenoids may be beneficial for preventing or reducing the severity of cardiac fibrosis.

- Astaxanthin: A reddish-orange aquatic carotenoid, found in salmon, red snapper, and shrimp, was able to reduce cardiac fibrosis in a rat model of pressure overload-induced cardiac fibrosis, which was facilitated by the TGF-β1/Smad signaling pathway. The studies have also suggested that carotenoids may have anti-inflammatory and antifibrotic effects in various organs, including the heart [186]. More research, however, is required to completely comprehend the mechanisms underlying these effects and to determine whether carotenoid supplementation could be a useful strategy for preventing or treating cardiac fibrosis in humans.

#### 6.1.12. Omega-3 Fatty Acids

- Omega-3 fatty acids are a group of polyunsaturated fatty acids that are essential for human health and are commonly found in fish oil and certain plant sources such as flaxseed oil, chia seeds, and walnuts. These fatty acids are known for their anti-inflammatory properties and have been shown to have numerous cardiovascular benefits. They are already used for the treatment of hypertriglyceridemia, a cardiovascular risk factor. Although the OMEMI clinical trial [187] reported negative results regarding the cardiac benefits of omega-3 fatty acids [186], there is evidence suggesting that omega-3 fatty acids may be beneficial for preventing or reducing the severity of cardiac fibrosis. Nevertheless, this trial highlights the need for further studies to determine the clinical role of these molecules in preventing or treating cardiac fibrosis.
- Studies have shown that DHA (Docosahexaenoic acid) from microalgae had protective effects on diabetes-induced cardiac fibrosis through boosting fatty acid oxidation in cardiac fibroblast and maintenance of ECM homeostasis. In addition, cardiac fibroblasts and myofibroblasts’ increased apoptosis and autophagy, decreased proliferation, and transdifferentiation were primarily responsible for maintaining ECM homeostasis [188].
- Eicosapentaenoic Acid (EPA) may have anti-inflammatory and anti-ibrotic effects in the heart of Spontaneously Hypertensive Rats. Such effects of EPA are facilitated in part by polarization of macrophages toward anti-inflammatory M2 phenotype and enhancement of the anti-inflammatory cytokine, IL-10 [189].

#### 6.1.13. Bioactive Peptides

Bioactive peptides are short chains of amino acids derived from proteins and have specific biological functions in the body [127,190]. Studies have investigated the potential benefits of bioactive peptides derived from milk proteins, such as casein and whey proteins. These peptides may have antihypertensive, anti-inflammatory, and antioxidant effects that could also be beneficial for cardiovascular health and the prevention of fibrosis.

- DIKTNKPVIF peptide from potatoes may be utilized to treat hypertensive myocardial damage. Exercise and DIKTNKPVIF peptides work together synergistically to reduce myocardial damage induced by spontaneous hypertension. This may be accomplished via modulating the AMPK/SirT1/PGC-1/FOXO3 energy metabolism signaling pathway and preventing myocardial fibrosis [191].
- IF and DI peptides extracted from potatoes had a therapeutic benefit in protecting against cardiac inflammation, hypertrophy, and fibrosis under hypertensive conditions. This occurs by downregulating protein expression of the hypertrophy markers especially BNP and MYH, the major inflammatory markers such as p-NFkB, IL-6, and TNF-α, and the fibrotic markers such as TIMP1, CTGF, uPA, and MMP-2 [192].
- Another study on moth bean seeds bioactive peptide revealed it has an ACE inhibitory properties. It effectively reduced systolic blood pressure, cardio–renal hemorrhage, and fibrosis in dexamethasone-induced hypertensive rats [193].
- Similar results were also observed in chicken muscle hydrolysate, which reduced fibrosis in spontaneously hypertensive rats [194].

While the research on bioactive peptides and cardiac fibrosis is still in its early stages, there is growing evidence to suggest that certain peptides may have potential benefits for cardiovascular health. However, more investigation is required to fully comprehend the mechanisms underlying these benefits and to establish the ideal bioactive peptide doses and sources for the prevention or treatment of fibrosis. Additionally, the development and use of peptides as therapeutic agents can be complex and expensive, and there are currently no peptide-based therapies approved for the treatment of cardiac fibrosis.

#### 6.1.14. Vitamin D

Vitamin D is a fat-soluble vitamin found in fatty fish, egg yolks, and fortified dairy products. Vitamin D deficiency has been linked to an increased risk of cardiovascular disease, and vitamin D administration may help prevent cardiac fibrosis by decreasing inflammatory reactions and TGF-β expression in the heart [195,196]. However, it is important to note that clinical trials investigating the cardiovascular benefits of vitamin D supplementation have shown an overall lack of significant results [197]. This raises questions about the potential impact of vitamin D on cardiac fibrosis. Further research is needed to clarify the relationship between vitamin D supplementation, cardiovascular health, and its specific effects on cardiac fibrosis.

#### 6.1.15. Probiotics

Probiotics are live bacteria and yeasts that are found in certain foods, such as yogurt and kefir, and are also available as dietary supplements. Studies have shown that certain probiotics can reduce inflammation and improve cardiac function, which may help prevent or treat cardiac fibrosis. Chao et al. have also shown that multistrain oral probiotic administration prevents obesity-induced heart fibrosis [198]. Another report by Wang et al. showed that *Bifidobacterium pseudolongum* and *Clostridium butyricum* attenuate the development of cardiac fibrosis in mice [199]. However, it is important to note that the topic of probiotics is complex as they can impact the gut microbiome, and an increasing number of studies are exploring their role in various disease states [200]. Further research is necessary to fully understand the mechanisms by which probiotics influence cardiac fibrosis and their overall effects on the gut microbiome in the context of different disease conditions.

**Table 2:** Reported Bioactive against cardiac fibrosis (Figures were adopted and modified from ACS, MDPI, Sigma, and Pepdraw).

Bioactive	Class	Structure	Source	Reference
Quercetin	Flavonoids	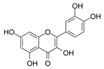	Onions, apples, and broccoli	[134,136]
Epicatechin	Flavonoids	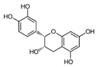	Cocoa, dark chocolate, red wine and tea	[137]
Kaempferol	Flavonoids	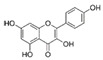	Apple, grape, tomato, green tea, broccoli, pine, and ginkgo leaf.	[138]
Apigenin	Flavonoids	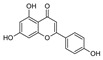	parsley, celery, basil, chamomile, cilantro, and oregano	[140]
Allicin	Organosulfur compounds	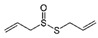	Garlic	[142,143]
Sulforaphane	Organosulfur compounds	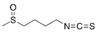	Broccoli and Brussels sprouts.	[144,145,146]
Allitridin (Diallyl trisulfide)	Organosulfur compounds	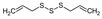	Garlic	[147]
S-allylcysteine	Organosulfur compounds	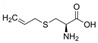	Garlic	[148,149]
β-Caryophyllene	Terpenoids	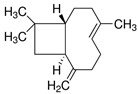	many herbs and spices, including black pepper, oregano, and cloves	[151]
Limonene	Terpenoids	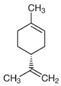	citrus fruits, including oranges, lemons, and limes	[152,153]
Ursolic acid	Terpenoids	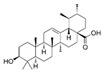	Apples, bilberries, cranberries, elder flower, peppermint, lavender, oregano, thyme, hawthorn, and prunes	[154,155]
Rosmarinic acid	Terpenoids	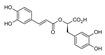	Culinary herbs such as lemon balm, rosemary, oregano, sage, thyme, and peppermint.	[156]
Resveratrol	Phenols	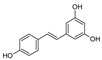	Peanuts, berries, and grapes.	[158,159,160,161,162]
Curcumin	Phenols	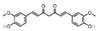	*Curcuma longa* L. (turmeric) rhizome	[163,164,165,166,201]
Ellagic acid	Phenols	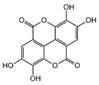	blackberries, raspberries, strawberries, cranberries, walnuts, pecans, pomegranates, and wolfberry,	[167]
Berberine	Alkaloids	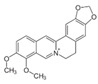	European barberry, goldenseal, goldthread, Oregon grape, phellodendron, and tree turmeric	[167]
Vincamine	Alkaloids	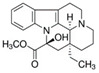	leaves of Vinca minor (lesser periwinkle)	[168,169]
Piperine	Alkaloids	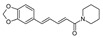	black pepper (Piper nigrum)	[171,202]
Ginsenoside	Saponins	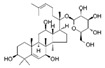	Ginseng root	[173]
Astragaloside IV	Saponins	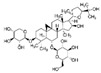	Astragalus membranaceus	[172]
Coumarins	Coumarins	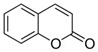	tonka bean, cassia bark	[174]
Stilbenes	Stilbenes	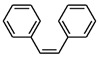	grapes, peanuts, and berries	[150,177]
Caffeic acid	Phenolic acids	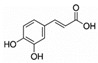	apples and red wine	[178,179]
Chlorogenic acid	Phenolic acids	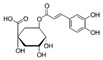	Coffee	[180,181]
Vanillic acid	Phenolic acids	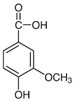	vanilla beans	[182]
Anthocyanins	Anthocyanins	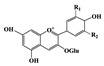	red and purple berries, grapes, apples, plums, cabbage, or foods containing high levels of natural colorants	[183]
Tannins	Tannins	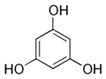	plant-based foods and beverages, such as tea, coffee, and wine	[184]
Astaxanthin	Carotenoids	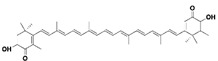	algae, yeast, salmon, trout, krill, shrimp, and crayfish	[186]
Docosahexaenoic acid	Omega-3 fatty acids	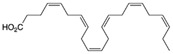	cold-water, fatty fish, such as salmon.	[188]
Eicosapentaenoic Acid	Omega-3 fatty acids	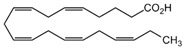	cold-water fatty fish, such as salmon.	[189]
DIKTNKPVIF	Peptides	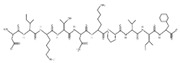	Potato	[191]
IF	Peptides	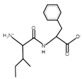	Potato	[192]
DI	Peptides	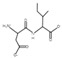	Potato	[192]
Vitamin D	Vitamins	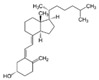	fatty fish, egg yolks, and fortified dairy products	[195,196]

### 6.2. Bioavailability of Bioactive Compounds

Bioavailability is an important factor to consider when using bioactive compounds as dietary supplements. Bioavailability refers to the extent and rate at which a compound is absorbed into the bloodstream and made available for use by the body. Many bioactive compounds have low bioavailability when consumed orally, meaning that only a small percentage of the compound is absorbed and utilized by the body. This can limit their effectiveness as dietary supplements. However, several factors must be considered to enhance the bioavailability of bioactive compounds, such as:

- Formulation: The form in which the bioactive compound is consumed can have a significant impact on its bioavailability. For example, some compounds may be more bioavailable in liquid or capsule form compared to tablets or powder [203].
- Molecular structure: The molecular structure of the bioactive compound can affect its ability to be absorbed and utilized by the body. Some compounds may be more readily absorbed if they are in a specific form or if they are combined with certain carriers or excipients [204].
- Interactions with other substances: Some substances in food or other dietary supplements can interact with bioactive compounds, affecting their bioavailability. For example, certain compounds may be more readily absorbed when consumed with a specific type of food or nutrient [205].
- Individual differences: Bioavailability can vary widely between individuals due to differences in genetics, gut microbiota, and other factors [206].

In order to improve the bioavailability of bioactive compounds, various strategies have been developed: (a) Formulating the compound in a more bioavailable form, such as a liposomal or nano-emulsion formulation. (b) Combining the compound with other substances that can enhance absorption, such as piperine (found in black pepper) or phospholipids. (c) Encapsulating the compound in a protective coating to prevent degradation in the digestive system [207]. (d) Using delivery systems such as transdermal patches or intravenous injections to bypass the digestive system altogether. By improving the bioavailability of bioactive compounds, it may be possible to enhance their therapeutic potential and improve their effectiveness as dietary supplements.

## 7. Future Perspective

Translation of antifibrotic strategies from bench to bedside in cardiac diseases is long and unpredictable. Accumulating evidence suggests that the regulatory networks governing fibrogenesis are tissue-specific [208]. Therefore, antifibrotic strategies should avoid generalizations and oversimplified views while considering the heterogeneity of the pathophysiology of myocardial diseases and the distinct portrait of fibrotic changes found in different types of myocardial insults. A pathophysiologic classification of the patients with heart failure is required to single out the subpopulations with exaggerated, inappropriate, progressive, or unconstrained fibrotic responses. These pathological groups might be perfect candidates for different antifibrotic approaches.

Studies have found a close association between the aberrant expression of different pathogenic genes and the development of cardiac diseases. Epigenetic and histone modifications play key roles in regulating the transcription of these genes following injury and ischemia. Although a large portion of the studies and many clinical trials have focused on the availability and role of various regulators of epigenetic modification enzymes, mostly on oncological diseases, some epigenetic inhibitors have shown to have potential responses in cardiovascular diseases [209]. The scope of these genetic and epigenetic interventions needs more focused research to discover potential targeted therapies for cardiac fibrosis. The reversible methylation modification profile aided by histone methyltransferases and demethylases has a pivotal role in multiple physiological and pathological processes that need further exploration.

Along with the genetic and epigenetic regulations, different metabolic, inflammatory, immunological, and molecular mechanisms collectively contribute to cardiac fibrosis. The shift from a normal metabolic state to the altered metabolic demand in the fibrosing microenvironment has created avenues of research in metabolomics in cardiac fibrosis. Moreover, the stressed up and dying myocardium in different fibrogenic conditions express different molecular patterns and attract inflammatory cells, ultimately changing the phenotype of normal fibroblasts. Focused and synchronized research is warranted in cardio-metabolomics and inflammation to halt or delay fibrosis progression. A coordinated antifibrotic approach that collectively targets crucial inflammatory signaling molecules, profibrotic cytokines, and cellular functions should be considered in developing therapies to treat fibrosis adequately and successfully.

Perhaps the use of drugs in combined therapy might prove effective in the treatment of cardiac fibrosis. The use of Spironolactone and Eplerenone, which have been shown to reduce myocardial fibrosis in animals, may be of great impact in human. However, more research and clinical trials are needed to confirm their beneficial effects and possible side effects. Other drugs currently in clinical trials such as vericiguat could be used in combination with other well-tolerated therapies such that the side effects of prolonged use could be abolished.

There is potential for optimizing the use of bioactive compounds for the prevention and treatment of cardiac fibrosis by combination of bioactive compounds with existing therapies for cardiovascular disease, such as ACE inhibitors and β-blockers, as these compounds are bioavailable and could easily be incorporated into diet. This approach may enhance the efficacy of existing therapies and improve clinical outcomes for patients with cardiac fibrosis and heart failure.

In addition, the use of novel delivery systems, such as nanoparticles and liposomes, improves the delivery of bioactive compounds to target tissues. These delivery technologies have the potential to improve the therapeutic potential of bioactive substances for the prevention and treatment of cardiac fibrosis. Furthermore, besides developing innovative delivery systems and coupling bioactive chemicals with established therapies, the potential negative effects and interactions of these compounds must be considered. While bioactive compounds have shown promise in preclinical studies, their safety and efficacy in humans need to be carefully evaluated through clinical trials. It is also important to consider the optimal dosing and duration of treatment for bioactive compounds. Some compounds may have dose-dependent effects, while others may exhibit toxicity at high doses. The duration of treatment may also be important, as some compounds may exhibit beneficial effects only after long-term treatment.

## Data Availability

Not applicable.
